# Long Conjugate Sequence Resistance Training to Improve Strength, Power and Competition Performance in Speed Skaters

**DOI:** 10.3390/jfmk11020186

**Published:** 2026-05-05

**Authors:** Froukje Sliedrecht, Kilian Stoker, Inge K. Stoter, Stein G. P. Menting, Marije T. Elferink-Gemser

**Affiliations:** 1 Center for Human Movement Sciences, University Medical Center Groningen, University of Groningen, 9712 CP Groningen, The Netherlands; f.sliedrecht@knsb.nl (F.S.); innovatielab@thialf.nl (I.K.S.); 2 Koninklijke Nederlandsche Schaatsenrijders Bond, 3565 CE Utrecht, The Netherlands; 3 Team NL Centrum Noord, 8444 AP Heerenveen, The Netherlands; k.stoker@topsportnoord.nl; 4 Innovation Lab Thialf, 8443 DA Heerenveen, The Netherlands; 5 Sport and Exercise Sciences Research Institute, Ulster University, Belfast BT15 1ED, UK

**Keywords:** longitudinal, physical conditioning, muscle physiology, athletic performance, speed skating

## Abstract

**Background**: Long conjugate sequence (LCS) resistance training aims to maintain and increase strength and power to enhance sport-specific performance. This study examined (1) how strength and power change during alternating periods of an LCS program, and (2) the relationship between strength, power, and performance in long-track (LT) and short-track (ST) speed skating. **Methods**: Twenty-three speed skaters (sixteen men, seven women; age = 18.0 [17.0, 19.0], sub-elite to elite performance level) followed a 39-week LCS program alternating strength and power periods. Strength (Isometric Mid-Thigh Pull) and power (Squat Jump) were assessed after each period. Linear mixed-effects models were used to examine the effects of time (baseline and four follow-up assessments), sport (long-track vs. short-track), and sex (male vs. female) on absolute and relative measures of strength and power. Models included fixed effects for time, sport, and sex with all two-way interactions, a random intercept for participants, and a first-order autoregressive covariance structure to account for repeated measures. Model selection followed backward elimination guided primarily by the corrected Akaike Information Criterion (AICc). Kendall’s tau correlations tested associations between strength, power, and competition performances closest to assessments. **Results**: Change in absolute and relative strength across the season differed by sex (F_(4, 33.57)_ = 2.72, *p* = 0.046; F_(4, 31.86)_ = 3.50, *p* = 0.02), with an increase only in male skaters (baseline to Test 4: +406 N ± 115, *p* = 0.01; +4.37 N/kg ± 1.41, *p* = 0.03). Absolute power changed (F_(4, 33.74)_ = 3.81, *p* = 0.01) specifically in the early season (baseline to Test 1: +151 N ± 40, *p* < 0.01), while relative power remained stable (F_(4, 53.05)_ = 1.94, *p* = 0.12). Higher absolute strength and power correlated with better LT (*n* = 12, τ = −0.58–0.67) and ST (*n* = 9, τ = −0.56–0.89) performance. Yet, relative strength related only to ST performance (*n* = 9, τ = −0.78–0.89), and relative power chiefly to the first 100 m of a LT performance (*n* = 12, τ = −0.49). **Conclusions**: The LCS method is associated with strength development without compromising power. The findings highlight the relationship between resistance training-induced outcomes and speed skating performance.

## 1. Introduction

Resistance training plays an integral role in an athletes’ pathway to optimize sports-specific performance [1]. It develops not only the capacity to generate relatively high forces against large resistances (i.e., strength), but also produces a high work rate (i.e., power) [2]. Maximal physiological adaptations from resistance training are achieved by following the principle of periodization, which involves the systematic, sequential and integrative scheduling of resistance training sessions [3,4,5]. While the tenets of periodization (e.g., variation and individualization) are well-established, there are a multitude of methods to periodize resistance training [6,7]. The Long Conjugate Sequence (LCS) method alternates focus on developing one performance characteristic (e.g., strength), while maintaining the other performance characteristic (e.g., power) through minimal training volume [6,7,8,9,10].

LCS is a structured strength-training approach where you rotate different exercises and training methods over a longer period (weeks to months) to develop multiple physical qualities at once. Although there are no scientific studies known to the authors that show that LCS training is superior to other methods, there is indirect evidence from broader areas of sport science that support the principles behind it, i.e., concurrent or conjugate periodization, exercise variation, undulating (non-linear) training, and training strength and power simultaneously. More specifically, the approach is informed by the concept of training residuals, whereby adaptations in different physical qualities decay at different rates when training emphasis is reduced [7,8,9]. This allows performance characteristics developed earlier in the sequence to be strategically maintained with a minimal stimulus while subsequent phases prioritize other qualities [6,7,9]. Such sequencing is intended to manage interference between competing neuromuscular adaptations, particularly in high-level athletes for whom simultaneous high-volume training of strength and power may compromise recovery and adaptation [6,9,10]. This method is considered to be particularly suitable for high-level athletes since it involves avoidance of stagnation [10]. To train and perform optimally during the competitive season, insight into the temporal structure of adaptations across long-term LCS use is highly relevant for trainers. It supports informed decisions about the content of training programs as training time needs to be balanced with recovery time wisely [10]. The LCS periodization method is based on the basic principles of periodization and studies investigating the adaptations of the endocrine system of the body. However, empirical research regarding the strength and power developments during the alternating periods within the program is lacking [10,11]. Insights into these developments throughout the LCS periodized resistance training program could help practitioners improve the efficiency of training to enhance the competitive success of athletes [4].

In evaluating the value of the LCS periodized resistance training program in improving sport-specific performance, it is essential to take into account the relationship between strength and power, and sport-specific performance. So far, most studies investigating resistance training have taken these relationships into consideration when making choices about the study design (i.e., the chosen resistance training program and the tests) [12]. However, these choices are often based on general knowledge regarding important performance characteristics to excel in a particular sport, rather than based on the relationship between the included test(s) to measure the effectiveness of resistance training and sport-specific performance [12]. Speed skating lends itself to exploring these relationships, since strength and power are considered to be important performance characteristics of a successful speed skater [13,14]. Strength and power gains are only meaningful when they contribute to the execution of sport-specific tasks such as force production, movement velocity, and technical actions relevant to performance. If improvements in strength and power are unrelated to sport-specific performance, substantial training time could be allocated to their development without resulting in meaningful performance gains. The sport can be split into two disciplines: long track (LT), a time trial competition on a 400 m track with distinct lanes for each skater, and short track (ST), a head-to-head competition on a 111 m track with tighter corners in which skaters can contest positions. Although the differences between disciplines impact the biomechanical and physiological performance characteristics needed to excel [15], resistance training is a key component of the training programs in both disciplines [16].

This study aims to investigate the value of the LCS periodized resistance training program and its relationship to sport-specific performance. The primary objective is to assess the changes in strength and power of high-level athletes during the alternating phases of the program. It is hypothesized that strength and power will increase following periods where the program specifically targeted the enhancement of each characteristic. Adaptations in strength and power are expected to vary substantially between individuals, but overall greater gains are anticipated in absolute compared to relative measures, as the participants are adolescent athletes undergoing growth and maturation. Early training years are primarily used to learn and refine movement patterns. Potential ceiling effects from prior training are therefore expected to be minimal, especially given that this is one of the participants’ first exposures to this type of program.

Additionally, given that the LCS method emphasizes the maintenance of strength and power through minimal volume, it is further hypothesized that strength and power will either remain stable or show improvement throughout the entirety of the resistance training program [17,18,19]. The secondary aim of this study is to investigate the relationship between strength, power, and sport-specific performance in speed skating. Given that strength and power are considered essential performance characteristics for successful speed skaters, it is hypothesized that both will exhibit a positive relationship with speed skating performance [13,20]. Specifically, strength is expected to be associated with counteracting centrifugal forces during tight cornering in ST speed skating [21], while power is anticipated to correlate with the ability to accelerate in both sports.

## 2. Materials and Methods

### 2.1. Participants

Ethical approval was granted by the University Medical Centre Groningen in accordance with the ethical standards of the Helsinki Declaration (ECB2017.01.09_1R1). Participants were Dutch junior speed skaters scouted for having the potential to reach podium places at international tournaments. All participants were above the age of 16 and trained at least six times a week, of which two were resistance training sessions. Before partaking, all participants were informed both verbally and by a written information letter about the aim, procedures and requirements of the study. Participants were included if they approved by signing the informed consent form and did not suffer from any musculoskeletal injury or medical condition that prevented them from executing the resistance training program. A total of 37 participants were recruited and all participants agreed to participate in the study. All participants met the inclusion criteria prior to participating in the study, yet fourteen were excluded from the analyses since they either did not complete the resistance training program as prescribed (*n* = 6), were insufficiently present at the strength and power assessments (<50% attendance, *n* = 5) or got an injury or medical condition (*n* = 3). Participants with <50% attendance at the strength and power assessments were excluded because these test outcomes constituted the primary indicators of training-induced adaptations, and insufficient test participation resulted in extensive missing outcome data, limiting the interpretability of longitudinal changes. The demographics of the 23 included participants can be found in Table 1. All included LT speed skaters were in the top 50 of their category on all distances in the KNSB ranking and were considered elite to sub-elite according to the performance benchmarks of Stoter et al. (2019) [22]. No junior rankings were available for the ST speed skaters.

### 2.2. Resistance Training Program

The participants followed a 39-week LCS periodized resistance training program from preseason to the competitive part of the season, divided into five periods. The preparatory period focused on enhancing the stability, mobility and general strength of the participants [23]. This phase primarily included bilateral and unilateral lower-body exercises (e.g., back squat and rear-foot elevated split squat), upper-body pushing and pulling exercises (e.g., weighted push-ups and rowing variations), and trunk stability exercises. This was followed by periods that alternately focused on improving strength or power. A complete overview of the training program can be found in Supplementary Materials Table S1.

Periods with a main emphasis on improving strength incorporated exercises with high loads, low to moderate movement velocities (i.e., maximum concentric velocity < 0.5 m/s or ranging from 0.5 to 0.75 m/s), performed with low repetition ranges (typically 3–6 repetitions per set) and longer inter-set rest intervals (approximately 120–180 s). These sessions predominantly included multi-joint resistance exercises targeting the lower body, complemented by upper-body pushing and pulling exercises and trunk stability work. Typical lower-body exercises included squat-based and hinge-based movements, with exercise variations progressing across periods while maintaining consistent movement patterns.

Periods focusing on increasing power incorporated similar movement patterns but were performed with moderate loads, a moderate to high movement velocity (i.e., maximum concentric velocity ranging from 0.75 to 1.0 m/s or from 1.0 to 1.3 m/s) [24,25,26,27,28,29,30,31], typically performed with moderate repetition ranges (approximately 4–8 repetitions per set) and rest intervals comparable to, or slightly shorter than, those used in strength-focused sessions.

Each week included two resistance training sessions of approximately 90 min each in which variations in different strength and power exercises were performed. Across phases, exercise selection was progressed through variations in the same fundamental movement patterns rather than through entirely novel exercises. Training loads were individualized based on maximum concentric velocity. LT and ST skaters followed similar programs, with minimal variations in frequency resulting from the scheduling of competitions. Velocities were monitored during training using the GymAware Linear Positional Transducer (GymAware; Kinetic Performance Technologies, Canberra, Australia) [12]. Adherence to the training program and proper execution of exercises were monitored in person by the strength and conditioning coach of the athletes.

### 2.3. Testing Procedures

Tests were scheduled around the end of the five periods (Table 2). Due to the elite sports environment, testing could not always be conducted at the exact end of each period. For example, scheduling had to take into account changes in the training program due to load monitoring or competition schedules. The exact timing of each assessment relative to the training phases and competitions is shown in Table 2.

**Figure 1:**
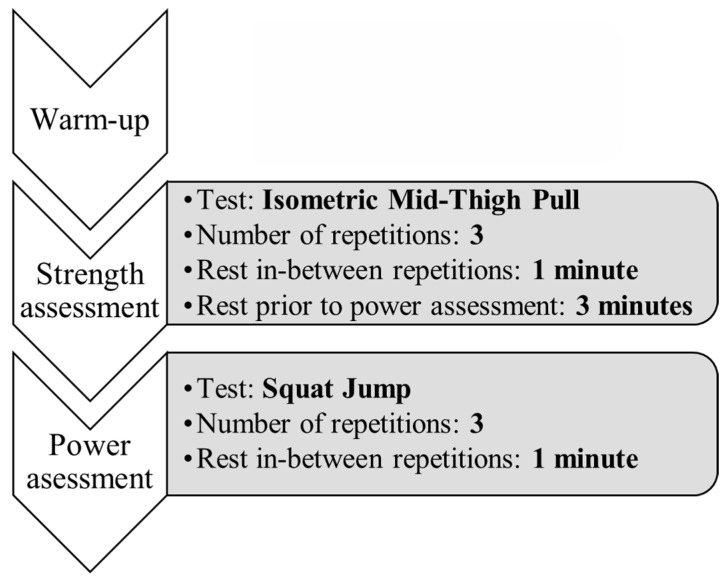
Overview of the testing procedures.

Participants refrained from any strenuous exercise for a minimum of 12 h before testing. All tests were performed barefoot on a dual force plate system (ForceDecks Lite, VALD, Brisbane, Australia) at the resistance training facilities of Thialf, Heerenveen. The force plates were positioned at a fixed location with a two centimeters gap in-between to minimize interference, and calibrated prior to the testing procedures. Data were gathered through VALD ForceDecks Jump version 2.0.8000 and analyzed using VALD ForceDecks version 2.0.8.000. Tests were conducted in a standardized order, starting a generalized warm-up, three repetitions of the strength assessment, followed by three minutes rest and three repetitions of the power assessment. An overview of the testing procedures can be found in Figure 1. Participants were familiar with all testing procedures and were verbally encouraged to give maximal effort through all attempts.

#### 2.3.1. Strength Assessment

The Isometric Mid-Thigh Pull (IMTP) is a valid and reliable test to measure maximum lower-body strength [32,33]. The closed-chain isometric test requires the participant to pull with a maximal effort on a fixed barbell at the height of their mid-thigh for three to five seconds. The participants performed three repetitions with one minute rest in-between. Absolute strength was defined as the highest force achieved during the test trial. Relative strength was defined as the absolute strength in newtons divided by the participant’s body mass in kilograms. The repetition with the highest absolute and relative strength was used for analysis.

#### 2.3.2. Power Assessment

The Squat Jump (SJ) is a valid and reliable test to measure explosive lower-limb power [34]. During the test, the participant descends to a squat position with the hands placed on the hips and pauses for two seconds before initiating a maximal vertical jump. No arm or leg swings were allowed in the air. Participants performed three repetitions of the SJ with one minute rest in-between. Absolute power was defined as the highest power output (Watt) during the push-off phase of the SJ. Relative power was defined as the absolute power (Watt) divided by the participant’s body mass in kilograms. Absolute power and relative power were averaged for the three repetitions.

### 2.4. Speed Skating Performance

For LT participants, performance was quantified as the time in seconds needed to cover 100 m (split time) and 500 m (total time) during the competition closest to the week 24 power and strength assessments (Table 2). Data were gathered from the KNSB. Competition times were excluded from the analyses if: (i) they preceded or followed the testing procedures by more than 10 days or less than 12 h, or (ii) they were not skated at the indoor ice rink of Thialf to control for potential effects of the ice rink or altitude on the performance [35]. For ST participants, performance was operationalized as the time in seconds needed to finish a 222 and 666 m time trial. These time trials took place in week 26 of the program at Thialf as part of the Dutch trials, during which speed skaters are selected to present The Netherlands at the first two ST World Cup competitions of the season (Table 2). The data are publicly available via ShorttrackOnline.info.

### 2.5. Statistical Analysis

Statistical analyses were performed using IBM SPSS Statistics for Windows version 28.0. Linear mixed modeling was used to examine the effects of time (baseline and four subsequent test moments), sport (long-track vs. short-track), and sex (male vs. female) on absolute strength, relative strength, absolute power, and relative power. For each variable, the initial model included fixed effects of time, sport, and sex, along with all two-way interactions. A random intercept was specified for each participant to account for between-subject variability. Repeated observations across time were modeled using a first-order autoregressive covariance structure (AR1).

Models were estimated using restricted maximum likelihood with Satterthwaite approximation for degrees of freedom. Model selection followed a backward-elimination procedure in which non-significant interaction terms were removed when their removal improved model fit. All information criteria reported by SPSS (−2 Log Likelihood, AIC, AICc, and BIC) were inspected during model comparison, with AICc used as the primary decision criterion due to the relatively small sample size. For each outcome, the model with the lowest AICc and acceptable interpretability was retained as the final model.

Estimated marginal means were derived for significant main or interaction effects involving time. Pairwise comparisons were restricted to contrasts between successive test moments, as well as between baseline and the final test. A Bonferroni adjustment was applied to control for multiple comparisons (α_adjusted = 0.05/5) [36]. Statistical significance was set at *p* < 0.05 (adjusted where applicable).

Kendall’s tau correlation coefficients were calculated between absolute strength, relative strength, absolute power, and relative power at Test 3, and LT and ST speed skating performance. Negative correlations indicate that a higher score on the strength and power assessments is related to a shorter time to cover a certain distance, indicating better speed skating performance, and vice versa. Correlations were interpreted as small (τ > 0.07), medium (τ > 0.20), or large (τ > 0.34) [37,38]. The two-sided significance level was set at *p* < 0.05.

## 3. Results

Absolute strength, relative strength, absolute power and relative power over the course of the resistance training program can be found in Table 3. Across the included sample, 11.7% of the strength and power assessment data were missing.

**Figure 2:**
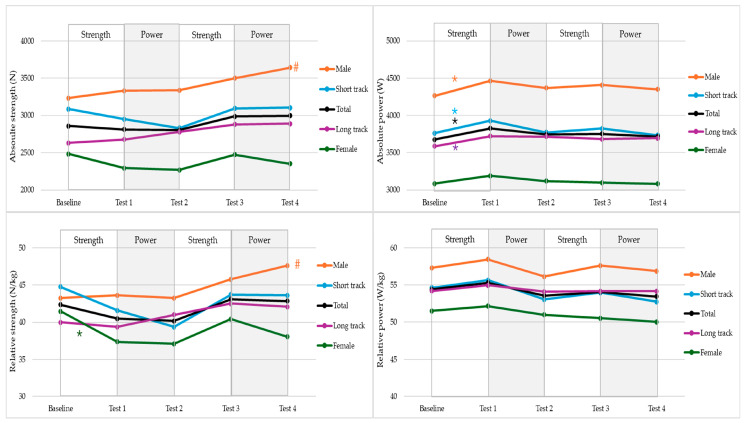
Estimated marginal means for absolute and relative strength and power across five tests, presented by sex and sport. # = significant difference between baseline and Test 4 (*p* < 0.05), * = significant difference between successive tests (*p* < 0.05).

Model estimates for power and strength can be found in Table 4 and Table 5, respectively. Estimated marginal means and results of the planned pairwise comparisons are graphically represented in Figure 2.

When controlling for time and sport, all outcome variables were estimated to be significantly higher in males compared with females. After adjusting for sex and sport, absolute strength did not change significantly across the study period (F_(4, 34.51)_ = 1.91, *p* = 0.13). Yet, when separated by sex, there was a significant change over time (F_(4, 33.57)_ = 2.72, *p* = 0.046). Planned pairwise comparisons indicated that, when controlling for sport, male skaters demonstrated a significant increase in absolute strength between baseline and the final test (Δ = 406 N ± 115, *p* = 0.01, 95% CI: –648 to –164).

When controlling for sport and sex, relative strength changed significantly over time (F_(4, 2.85)_ = 33.41, *p* = 0.04). Pairwise comparisons showed a significant increase between Test 2 and Test 3 (Δ = 2.92 N/kg ± 1.03, *p* = 0.03, 95% CI: –0.86 to 4.98). Additionally, there was a significant interaction effect between time and sex (F_(4, 31.86)_ = 3.50, *p* = 0.02). When controlling for sport, male skaters showed a significant improvement from baseline to Test 4 (Δ = 4.37 N/kg ± 1.41, *p* = 0.03, 95% CI: –1.41 to 7.32). In contrast, female skaters demonstrated a decrease from baseline to Test 1 (Δ = –4.08 N/kg ± 1.53, *p* = 0.05, 95% CI: –7.16 to –1.01).

After adjusting for sex and sport, absolute power changed significantly across the study period (F_(4, 33.74)_ = 3.81, *p* = 0.01). Specifically, absolute power increased between baseline and Test 1 (Δ = 151 N ± 40, *p* < 0.01, 95% CI: 70–232). When controlling for sport, male skaters demonstrated a significant increase from baseline to Test 1 (Δ = 200 N ± 44, *p* < 0.01, 95% CI: 111–288). Both long-track and short-track skaters exhibited increases from baseline to Test 1 (Δ = 135 N ± 48, *p* = 0.04, 95% CI: 38–231; and Δ = 167 N ± 61, *p* = 0.045, 95% CI: 45–290, respectively).

After adjusting for sex and sport, relative power did not change significantly across the study period (F_(4, 53.05)_ = 1.94, *p* = 0.12). None of the planned pairwise comparisons between time-points reached statistical significance.

Two LT speed skaters did not participate in a competition fitting the inclusion criteria, and were excluded from correlation analysis between absolute strength, relative strength, absolute power and relative power (at Test 3), and speed skating performance. For LT speed skaters, there was a large negative correlation between absolute strength and 100 m (τ = −0.58, *n* = 12, *p* < 0.01) and 500 m performance (τ = −0.64, *n* = 12, *p* < 0.01), meaning a higher strength score is associated with a faster time. There was no significant association with relative strength (τ = −0.30, *n* = 12, *p* = 0.17; τ = −0.24, *n* = 12, *p* = 0.27). There were large negative correlations between absolute and relative power and 100 m performance (τ = −0.67, *n* = 12, *p* < 0.01; τ = −0.49, *n* = 12, *p* < 0.05), and a large negative correlation between absolute power and 500 m time (τ = −0.67, *n* = 12, *p* < 0.01). No significant association was found between relative power and 500 m time (τ = −0.36, *n* = 12, *p* = 0.10).

As for ST speed skaters, there was a large negative correlation between absolute and relative strength and 222 m (τ = −0.83, *n* = 9, *p* < 0.01; τ = −0.89, *n* = 9, *p* < 0.001) and 666 m time (τ = −0.78, *n* = 9, *p* < 0.01; τ = −0.83, *n* = 9, *p* < 0.01), respectively. Furthermore, a large, significant, negative correlation was found between absolute power and performance in the 222 m trial (τ = −0.56, *n* = 9, *p* < 0.05), but not the 666 m trial (τ = −0.50, *n* = 9, *p* = 0.06). No significant correlations were found between relative power and 222 m (τ = −0.44, *n* = 9, *p* = 0.10) or 666 m time (τ = −0.28, *n* = 9, *p* = 0.30).

## 4. Discussion

This study is the first to investigate changes in strength and power during an LCS periodized resistance training program in high-level athletes. By following athletes across the entire season, it provides a comprehensive overview of how strength and power develop from the preseason through to the competitive phase. The findings demonstrate the key advantage of the LCS method, namely the maintenance of both strength and power during training blocks where these qualities were not the primary focus. What is unique in the present study is that this was observed in highly trained athletes across a full season of competition, a context in which the preservation of strength and power cannot be taken for granted. Across this period, absolute and relative strength increased, specifically in male skaters, while relative and absolute power were maintained. Furthermore, it was demonstrated that particularly absolute strength and power correlated with LT and ST speed skating performance. These findings address a notable gap in the literature and provide coaches and athletes with practical insights to optimize training efficiency and, ultimately, competitive success.

### 4.1. Strength and Power Change During a LCS Periodized Resistance Training Program

When analyzing absolute and relative strength during the alternating periods of the resistance training program, varying results were found. The overall absolute and relative strength increase over the study period seems to be driven mainly by increases in male athletes. Although absolute and relative power did not increase or decrease over the whole competitive season, there was a significant increase in absolute power between the baseline and first test.

These results show the hybrid effect of LCS interaction with a specific sport. It might appear contradictory to the original hypothesis, as it was expected that strength would increase after periods with a main emphasis on improving strength, based on previous research showing that periods with a main emphasis on high loads are beneficial in increasing strength [24,29,31]. However, strength development follows a non-linear time course, reflecting the interaction between neuromuscular adaptation, fatigue accumulation, and the residual effects of prior training [6,7,8,28,39,40]. In this context, strength gains observed during power-focused periods may represent a delayed realization of adaptations induced during preceding strength periods, facilitated by reduced fatigue and partial maintenance of strength stimuli. Such responses resemble taper-like effects, where performance improvements emerge following a reduction in training load rather than during the highest loading phases [10,41]. The results show that with LCS it is possible to build strength over a longer period of time, also when combining with power focused periods. Further research is needed to get further insights into the determinants affecting the duration of the delay of musculoskeletal adaptations, for practitioners to take into account when periodizing resistance training.

As noted, there were no significant differences in relative power across the study period, and the change in absolute power was limited to an increase between the baseline and the first test. These results conflict with the hypothesis, which was based on previous research which demonstrated resistance training using moderate loads to be effective in increasing power [17,18,30,31]. There may be several explanations for these results. When benchmarked against the percentiles proposed by Centeno-Prada et al. [42], the relative power at baseline corresponded to the 75th percentile for women and the 90th percentile for men, indicating that the speed skaters demonstrated above-average relative power. The absence of an increase in power of the competitive season might be attributable to the law of diminishing returns, which states that the same training volume leads to progressively less progress as the baseline level increases [43]. In addition, improvements in maximal strength during earlier phases may have shifted the optimal load for power expression, potentially reducing the effectiveness of subsequent power-oriented blocks if loading strategies were not fully re-adjusted [24,25,26]. In this context, power development may require more narrowly focused or block-based strategies to elicit detectable adaptations beyond maintenance in high-level athletes [9,40]. Furthermore, the training program of the speed skaters contained training types other than resistance training. These strains on the speed skaters’ bodies might have affected the power changes during the program.

### 4.2. The Relationship of Strength and Power with Sport-Specific Performance in Speed Skating

Generally, strong relationships were found between absolute strength and power and LT and ST speed skating performance. These results are consistent with the hypothesis and previous research showing that absolute strength and power are important performance characteristics of a successful speed skater in both disciplines [13,20]. Still, when comparing both disciplines, the results show that in ST especially the capacity to generate relatively high forces against large resistances (i.e., strength) contributes to performance. This reflects the high centrifugal forces which need to be counteracted when racing at high speed through the tight corners. In LT especially, the capacity to produce a high work rate (i.e., power) is key. When comparing the first part of the time trial with the overall performance, it becomes clear that most variables correlate more strongly with the start than with the final time in both ST and LT. This aligns with the kinematic demands of start acceleration versus steady-state technique. Still, it needs to be acknowledged that the differences are small. This may be explained by the short duration of the time trials, which are considered sprints in both LT (500 m) and ST (666 m). Greater differences are expected with increasing skating distances, such as 1000 m, 1500 m, and 3000 m.

Considering LT speed skating performance, the results indicated that absolute strength and power are associated with performance in the first 100 m and the total 500 m. Yet, insignificant relationships were found between relative strength and power and LT speed skating performance, except for relative power at the start (first 100 m). These findings suggest that being somewhat heavier may not necessarily have to result in worse performance on the 500 m in LT speed skating. These findings are in line with the statements of multiple World Champion in the LT sprint distances (500 m and 1000 m) Jutta Leerdam, who narrated that being heavier made her stronger and more stable throughout the season [44].

ST speed skating performance was found to be associated with absolute and relative strength, while the relationships with absolute and relative power were slightly weaker. These findings could be explained by the high amount of tight cornering in ST speed skating, for which counteracting the centrifugal forces is a prominent aspect. For that reason, strength is considered to be an important performance characteristic of successful ST speed skaters [14]. Although regular ST speed skating competitions are won by competing against each other rather than time, the current study measured ST speed skating performance during a time trial in order to be able to objectively operationalize ST speed skating performance. It is, therefore, expected that the results of the current study are an underestimation of the relationship between regular ST competition performance and absolute strength, as regular competitions involve overtaking other ST speed skaters during the race, during which you cannot choose an ideal line in the corners requiring additional strength.

Not only the choice of a time trial as a speed skating performance measure in ST may have influenced the results, but also the choice for the strength and power assessment, i.e., the Isometric Mid-Thigh Pull (IMTP) and the Squat Jump. The execution of these tests is in a vertical direction whereas speed skating requires a lateral/diagonal push-off on the ice to produce forward velocity. Unfortunately, at this moment reliable and valid measurements for lower-limb strength and power production in a lateral direction and with alternating legs, like in speed skating, are lacking. As a consequence, it is expected that the relationships of strength and power with sport-specific performance in both ST and LT performance may have been even more pronounced when applying more ecological valid measures. However, this hypothesis needs empirical testing before conclusions can be drawn. Until then, IMTP and SJ are considered appropriate alternatives based on their frequently reported and accepted reliability and validity for measuring lower-body strength and power.

### 4.3. Practical Implications

The LCS periodized resistance training program is a valuable addition to the training program of athletes, such as speed skaters, who aim to increase strength throughout the season while preserving power. In contrast, when peak power development is the primary objective, such as during short preparatory phases or in sports where explosive power expression dominates performance outcomes, the LCS approach may be less suitable. Therefore, practitioners are recommended to gain insights into the importance of strength and power development or maintenance when selecting an appropriate periodization strategy.

The strong relationships between strength and power and sport-specific performance in LT and ST speed skating highlight the general importance of incorporating resistance training in the training program of speed skaters. However, speed skaters typically perform high volumes of sport-specific training, and the inclusion of heavy resistance training loads may increase fatigue and interfere with technical and metabolic demands if not appropriately managed. In this context, an additional factor that warrants consideration is the concept of the residual training effect, which pertains to the persistence of physiological and performance adaptations induced by systematic training stimuli for a defined period after the cessation of training. Strength is generally retained for a comparatively longer duration (approximately 30 ± 5 days), whereas power tends to decline after roughly one week (5 ± 3 days) [39,40].

Finally, the implementation of LCS facilitates the preservation of both qualities. Lastly, the current study provides an insight into the strength and power of high-level speed skaters assessed using the IMTP and SJ respectively, which are commonly used tests in practice. Therefore, the test results can be used as reference values for high-level speed skaters aged 16 to 21 years old. When doing so, practitioners should keep in mind the proportion of men and women within the groups.

### 4.4. Limitations

The study was conducted within high-level athletes who used resistance training as a means to improve sport-specific performance. As a result, the athletes engaged in competitions as well as training types other than resistance training during this study, such as speed skating and cycling. This inevitably causes strain on the body of the athletes which may have influenced the strength and power changes during the periods within the LCS periodized resistance training program in either a negative or positive way. Consequently, precise quantification of, and correction by, overall training load across the entire season was not feasible. Furthermore, this led to the necessity to be flexible in the timing of testing, resulting in the assessments not consistently aligning with the end of each training period. Although these effects can be seen as a limitation, they also come with a high ecological validity since the majority of high-level athletes has to deal with strains from training types other than resistance training. Secondly, the absence of a control group does not allow to directly attribute the changes in strength and power to the LCS periodized resistance training program. Unfortunately, the absence of a control group is inherent to studies in the high-level sports environment due to limited money, facilities and staff availability. Furthermore, given the high priority that high-level athletes place on improving sports performance, it is unethical to divide athletes of equal performance level into an experimental and control group as this may give one group an advantage over the other. Thirdly, there was a considerable amount of missing data in the current study. Finally, it should be kept in mind that the current study used a cross-sectional design. Therefore, the results of the current study give an insight into the relationship between strength and power and sport-specific performance, rather than indicating cause and effect [2]. Aside from strength and power, there are many aspects, such as tactical and technical factors, that determine successful sports performance [45]. Therefore, strength and power increases do not necessarily translate into better sports performance. Hence, further research is proposed to get an insight into the transfer and contribution of the strength and power gains as a result of the LCS periodized resistance training program to sport-specific performance.

## 5. Conclusions

The current study is one of the first to give insights into the strength and power changes in high-level athletes over the course of a LCS periodized resistance training program. Over the course of the program, absolute and relative strength increased, specifically in male skaters, and relative and absolute power were as maintained. In LT speed skaters, 500 m performance was primarily associated with absolute strength and absolute power, whereas 100 m performance was also associated with relative power. In ST speed skaters, performance was strongly associated with both absolute and relative strength, whereas absolute power was associated particularly with sprint performance (222 m). Therefore, when considered in relation to the sport-specific task requirements of LT and ST speed skating, the LCS periodized resistance training program appears to align with performance-relevant strength and power characteristics in speed skaters, although may be less suitable when specifically aiming to improve the start of the 500 m in LT speed skating. Further research is proposed to study the absence of power increases during the program, and could focus on extending the knowledge of the effectiveness of the LCS periodization method in increasing strength and power in athletes of different sports.

## Figures and Tables

**Table 1:** Baseline demographics.

Characteristic ^a^		Long Track (*n* = 14)	Short Track (*n* = 9)	Total (*n* = 23)
Sex ^a^	(m/f)	9/5	7/2	16/7
Age ^b^	(years)	18.0 [16.8, 18.3]	19.5 [18.0, 20.5] *	18.0 [17.0, 19.0]
Body mass	(kg)	67.0 [59.6, 76.8]	74.0 [68.2, 77.5]	72.2 [64.0, 76.2]
Skating experience	(years)	13.0 [10.3, 13.8]	14.0 [12.0, 14.5]	13.0 [11.0, 14.0]
RT ^c^ experience	(years)	2.0 [1.0, 2.0]	3.0 [2.0, 4.5] *	2.0 [2.0, 3.0]
100 m/222 m time	(seconds)	10.7 [10.4, 11.0]	20.6 [20.3, 21.5]	
500 m/666 m time	(seconds)	38.4 [37.0, 40.1]	57.1 [56.6, 60.4]	

^a^ number of male/female participants; ^b^ expressed as median [quartile 1, quartile 3]; ^c^ RT = resistance training; * *p* < 0.05 when comparing LT and ST.

**Table 2:** Overview of the study design.

Period ^a^	Preparatory						Strength					Power							Strength						Power														
Main focus ^b^	Preparatory Exercises ^c^						<0.5 m/s					0.75–1.0 m/s							<0.5 m/s						1.0–1.3 m/s														
Secondary focus	Prehab ^d^						0.5–0.75 m/s					<0.5 m/s							0.75–1.0 m/s						0.75–1.0 m/s														
Third focus	-						Prehab					Prehab							Prehab						< 0.5 m/s														
Week ^e^	1	2	3	4	5	6	7	8	9	10	11	12	13	14	15	16	17	18	19	20	21	22	23	24	25	26	27	28	29	30	31	32	33	34	35	36	37	38	39
Tests LT ^f^				B							1								2					3															4
Tests LT (rel. period end)				−2							0								+1					−1															0
Tests ST						B						1						2							3											4			
Tests ST (rel. period end)						0						+1						0							+1											−3			

^a^ Period definition within this study; ^b^ the main, secondary and third focus of the period was on performing exercises that incorporate loads that can be lifted at a maximum voluntary velocity as indicated; ^c^ exercises that aimed to get the athlete ready to train by enhancing the stability, mobility and general strength of the athlete; ^d^ preventative rehabilitation exercises which aimed to reduce the risk of injury; ^e^ week number counted from the start of the resistance training program; ^f^ the weeks in which the tests (B = baseline, 1 = Test 1, 2 = Test 2, etc.) were conducted for the long-track (LT) and short-track (ST) speed skaters are indicated in black. The week in which competition performance was retrieved is indicated in grey shading.

**Table 3:** Descriptive statistics, absolute and relative strength and power (sample size, mean, standard deviation), presented by sport, sex and test.

			Baseline			Test 1			Test 2			Test 3			Test 4		
			*n*	Mean	SD	*n*	Mean	SD	*n*	Mean	SD	*n*	Mean	SD	*n*	Mean	SD
**Absolute**	LT	Male	8	2898	493	8	3151	319	9	3224	393	9	3317	403	8	3495	375
**strength**		Female	5	2413	345	4	2198	509	5	2319	176	5	2423	299	5	2328	223
**(N)**	ST	Male	6	3676	500	6	3619	500	3	3070	252	7	3677	541	6	3867	537
		Female	2	2474	129	2	2442	238	2	2265	175	1	2491	-	2	2404	343
**Relative**	LT	Male	8	38.79	4.60	8	41.30	3.97	9	42.56	4.99	9	43.90	5.34	8	45.26	5.60
**strength**		Female	5	41.45	4.29	4	37.20	6.91	5	39.34	1.78	5	40.81	4.69	5	38.96	2.91
**(N/kg)**	ST	Male	6	48.04	6.82	6	46.10	5.91	3	40.41	0.55	7	47.54	5.37	6	50.69	4.92
		Female	2	40.37	3.50	2	38.40	1.12	2	35.24	1.65	1	42.51	-	2	37.12	2.06
**Absolute**	LT	Male	8	4221	515	8	4428	629	9	4374	606	9	4359	554	8	4480	410
**power**		Female	5	3008	425	4	3066	438	5	3038	400	5	3005	388	5	3062	438
**(W)**	ST	Male	6	4362	605	6	4566	603	2	3963	303	6	4452	596	6	4261	561
		Female	2	3131	0	2	3315	60	2	3262	125	1	3221	-	2	3091	156
**Relative**	LT	Male	8	56.70	6.02	8	57.87	7.22	9	56.49	7.33	9	57.45	6.28	8	58.05	6.05
**power**		Female	5	51.67	5.57	4	52.29	7.23	5	51.57	6.84	5	50.78	7.47	5	51.41	7.93
**(W/kg)**	ST	Male	6	56.74	5.28	6	57.96	4.57	2	55.00	8.20	6	56.50	4.89	6	56.00	6.94
		Female	2	51.28	7.11	2	52.39	5.69	2	50.88	4.36	1	54.97	-	2	48.13	6.63
**Body**	LT	Male	8	74.6	6.7	8	76.5	5.8	9	77.78	10.1	9	75.9	6.4	8	77.4	5.5
**weight**		Female	5	58.2	4.6	4	58.7	4.1	5	59.0	3.3	5	59.4	3.8	5	59.7	3.9
**(kg)**	ST	Male	6	76.6	4.2	6	78.5	4.8	2	72.4	5.3	6	78.6	5.6	6	76.2	5.8
		Female	2	61.7	8.6	2	63.7	8.1	2	64.5	8.0	1	58.6	-	2	64.6	5.7

Note: SD = standard deviation.

**Table 4:** Model estimates for absolute power and relative power.

	Absolute Power (W)				Relative Power (W/kg)			
	β (SE)	F (df)	*p*-Value	95% CI	β (SE)	F (df)	*p*-Value	95% CI
**Intercept**	3114 (36)	912.88 (1, 18.99)	<0.001	2357, 3872	49.00 (4.39)	1331.49 (1, 19.00)	<0.001	39.87, 58.14
**Time**		3.81 (4, 33.74)	0.01			1.94 (4, 53.05)	0.12	
Baseline	71 (108)			−157, 299	2.40 (1.54)			−0.72, 5.51
Test 1	190 (106)			−27, 407	3.15 (1.54)			0.04, 6.26
Test 2	44 (104)			−164, 252	1.15 (1.58)			−2.01, 4.32
Test 3	63 (90)			−119, 246	1.11 (1.47)			−1.83, 4.04
Test 4	(reference)				(reference)			
**Sport**	−63 (428)	0.25 (1, 18.98)	0.62	−956, 831	2.05 (5.15)	0.01 (1, 18.99)	0.92	−8.69, 12.79
**Sex**	1243 (410)	25.69 (1, 18.99)	<0.001	387, 2100	7.50 (4.95)	4.41 (1, 19.00)	0.049	−2.81, 17.81
Time * Sport		1.15 (4, 38.06)	0.35			0.73 (4, 55.46)	0.58	
Baseline * LT	−137 (99)			−345, 72	−1.83 (1.42)			−4.69, 1.03
Test 1 * LT	−169 (97)			−366, 28	−2.07 (1.41)			−4.90, 0.77
Test 2 * LT	−17 (64)			−220, 186	0.35 (1.56)			−3.47, 2.77
Test 3 * LT	−98 (79)			−257, 61	−1.23 (1.28)			−3.78, 1.33
Test 4 *ST	(reference)				(reference)			
Time * Sex		0.62 (4, 40.30)	0.64			0.67 (4, 44.25)	0.61	
Baseline * Male	−89 (102)			−303, 126	−1.05 (1.44)			−3.98, 1.87
Test 1 * Male	9 (101)			−197, 214	−0.54 (1.47)			−3.49, 2.41
Test 2 * Male	−17 (94)			−205, 172	−1.71 (1.43)			−4.56, 1.14
Test 3 * Male	46 (80)			−115, 207	0.24 (1.30)			−2.36, 2.84
Test 4 * Female	(reference)				(reference)			
Sport * Sex	45 (495)	0.01 (1, 18.94)	0.93	−992, 1082	−1.32 (5.94)	0.05 (1, 18.98)	0.83	−13.74, 11.11

Note: β: estimated marginal mean, SE: standard error, CI: confidence interval, df = degree of freedom, * indicates an interaction effect between variables, ST: short track, LT: long track.

**Table 5:** Model estimates for absolute and relative strength.

	Absolute Strength (N)				Relative Strength (N/kg)			
	β (SE)	F (df)	*p*-Value	95% CI	β (SE)	F (df)	*p*-Value	95% CI
**Intercept**	2357 (277)	1046.70 (1, 19.36)	<0.001	1786, 2928	37.18 (3.12)	1855.6 (1, 19.71)	<0.001	30.80, 43.55
**Time**		1.91 (4, 34.51)	0.13			33.41 (4, 2.85)	0.04	
Baseline	245 (214)			−204, 703	5.02 (2.60)			−0.48, 10.52
Test 1	−28 (199)			−434, 379	−0.35 (2.52)			−5.53, 4.83
Test 2	−164 (177)			−518, 191	−2.54 (2.38)			−7.30, 2.22
Test 3	122 (144)			−165, 410	2.20 (2.05)			−1.91, 6.32
Test 4	(reference)				(reference)			
**Sport**	−9 (317)	1.85 (1, 19.21)	0.19	−667, 648	1.76 (3.53)	0.70 (1, 19.55)	0.41	−5.51, 9.03
**Sex**	−1496 (308)	33.47 (1,19.34)	0.01	860, 2132	12.86 (3.43)	8.97 (1, 19.71)	0.01	5.82, 19.91
Time * Sport		−1.46 (4, 35.75)	0.24			2.01 (4, 34.98)	0.12	
Baseline * LT	−237 (194)			−647, 173	−3.23 (2.37)			−8.23, 1.76
Test 1 * LT	−58 (179)			−424, 309	−0.67 (2.29)			−5.35, 4.01
Test 2 * LT	165 (166)			−167, 496	3.19 (2.25)			−1.30, 7.67
Test 3 * LT	3 (125)			−246, 252	0.34 (1.77)			−3.20, 3.88
Test 4 *ST	(reference)				(reference)			
Time * Sex		2.72 (4, 33.57)	0.046			3.50 (4, 31.86)	0.02	
Baseline * Male	−538 (200)			−963, −112	−7.77 (2.44)			−12.94, −2.61
Test 1 * Male	−253 (188)			−636, 131	−3.34 (2.39)			−8.24, 1.55
Test 2 * Male	−221 (164)			−549, 106	−3.42 (2.19)			−7.81, 0.97
Test 3 * Male	−263 (130)			−523, −4	−4.22 (1.85)			−7.92, −0.52
Test 4 * Female	(reference)				(reference)			
Sport * Sex	−434 (357)	1.38 (1, 18.51)	0.26	−1154, 325		2.94 (1, 18.94)	0.10	−14.66, 1.46

Note: β: estimated marginal mean, SE: standard error, CI: confidence interval, df = degree of freedom, * indicates an interaction effect between variables, ST: short track, LT: long track.

## Data Availability

The data supporting the findings of this study are not available for public access or on request due to privacy and ethical restrictions. The small, specific nature of the cohort (elite Dutch junior speed skaters) makes the data highly susceptible to re-identification, even after anonymization.

## Supplementary Materials

The following supporting information can be downloaded at: https://www.mdpi.com/article/10.3390/jfmk11020186/s1, Table S1. Absolute strength (N), relative strength (N/kg), absolute power (W) and relative power (W/kg) of long-track (LT, *n* = 14), short-track (ST, *n* = 9), and the total group of participants (Total, *n* = 23) at baseline, Test 1, Test 2, Test 3 and Test 4.

## Abbreviations

The following abbreviations are used in this manuscript:

LCS	Long Conjugate Sequence
LT	Long Track
ST	Short Track
IMTP	Isometric Mid-Thigh Pull
SJ	Squat Jump
