# Supplementary material for: Long Conjugate Sequence Resistance Training to Improve Strength, Power and Competition Performance in Speed Skaters
Source: J Funct Morphol Kinesiol. 2026 May 5;11(2):186. doi: 10.3390/jfmk11020186 (PMC13214880; doi:10.3390/jfmk11020186)
Supplement: Supplementary file 1 [file jfmk-11-00186-s001.zip › jfmk-4034935-supplementary.pdf]

Preparatory phase

Day 1	week 1					week 2					week 3					week 4					week 5					Week 6				
Main Exercise	*Set*	*Reps*	*m/s*	*Rest*	*RIR*	*Set*	*Reps*	*m/s*	*Rest*	*RIR*	*Set*	*Reps*	*m/s*	*Rest*	*RIR*	*Set*	*Reps*	*m/s*	*Rest*	*RIR*	*Set*	*Reps*	*m/s*	*Rest*	*RIR*	*Set*	*Reps*	*m/s*	*Rest*	*RIR*
Lower body push	3	8		120 sec	5	3	7		120 sec	4	3	6		120 sec	3	3	5		120 sec	2 - 3	3	8		120 sec	5	3	7		120 sec	4
Lower body push : split stance	3	8 l/r		0 sec	5	3	7 l/r		0 sec	4	3	6 l/r		0 sec	3	3	5 l/r		0 sec	2 - 3	3	8 l/r		0 sec	5	3	7 l/r		0 sec	4
Upper body push	3	8		120 sec	5	3	7		120 sec	4	3	6		120 sec	3	3	5		120 sec	2 - 3	3	8		120 sec	5	3	7		120 sec	4
Lower body pull	3	8		0 sec	5	3	7		0 sec	4	3	6		0 sec	3	3	5		0 sec	2 - 3	3	8		0 sec	5	3	7		0 sec	4
Upper body pull	3	8		120 sec	5	3	7		120 sec	4	3	6		120 sec	3	3	5		120 sec	2 - 3	3	8		120 sec	5	3	7		120 sec	4
Accessory Exercise																														
Anti-lateral flexion	3	30 sec l/r		0 sec		3	40 sec L/r		0 sec		3	50 sec l/r		0 sec		3	60 sec l/r		0 sec		3	30 sec l/r		0 sec	5	3	40 sec L/r		0 sec	
Rotation	3	8 l/r		60 sec	5	3	8 l/r		60 sec	4	3	8 l/r		60 sec	3	3	8 l/r		60 sec	2 - 3	3	8 l/r		60 sec	5	3	8 l/r		60 sec	4

Day 2	week 1					week 2					week 3					week 4					week 5					week 2				
Main Exercise	*Set*	*Reps*	*m/s*	*Rest*	*RIR*	*Set*	*Reps*	*m/s*	*Rest*	*RIR*	*Set*	*Reps*	*m/s*	*Rest*	*RIR*	*Set*	*Reps*	*m/s*	*Rest*	*RIR*	*Set*	*Reps*	*m/s*	*Rest*	*RIR*	*Set*	*Reps*	*m/s*	*Rest*	*RIR*
Lower body push	3	8		120 sec	5	3	7		120 sec	4	3	6		120 sec	3	3	5		120 sec	2 - 3	3	8		120 sec	5	3	7		120 sec	4
Lower body push : split stance	3	8 l/r		0 sec	5	3	7 l/r		0 sec	4	3	6 l/r		0 sec	3	3	5 l/r		0 sec	2 - 3	3	8 l/r		0 sec	5	3	7 l/r		0 sec	4
Upper body pull	3	8		120 sec	5	3	7		120 sec	4	3	6		120 sec	3	3	5		120 sec	2 - 3	3	8		120 sec	5	3	7		120 sec	4
Lower body pull: hip (1 leg)	3	8 l/r		0 sec	5	3	7 l/r		0 sec	4	3	6 l/r		0 sec	3	3	5 l/r		0 sec	2 - 3	3	8 l/r		0 sec	5	3	7 l/r		0 sec	4
Upper body push	3	8		120 sec	5	3	7		120 sec	4	3	6		120 sec	3	3	5		120 sec	2 - 3	3	8		120 sec	5	3	7		120 sec	4
Accessory Exercise																														
Anti-extension	3	30 sec		0 sec		3	40 sec		0 sec		3	50 sec		0 sec		3	60 sec		0 sec		3	30 sec		0 sec		3	40 sec		0 sec	
Anti-flexion	3	30 sec		60 sec		3	40 sec		60 sec		3	50 sec		60 sec		3	60 sec		0 sec		3	30 sec		60 sec		3	40 sec		60 sec	

Strength phase 1

Day 1	week 1					week 2					week 3					week 4					week 5				
Main Exercise	*Set*	*Reps*	*m/s*	*Rest*	*RIR*	*Set*	*Reps*	*m/s*	*Rest*	*RIR*	*Set*	*Reps*	*m/s*	*Rest*	*RIR*	*Set*	*Reps*	*m/s*	*Rest*	*RIR*	*Set*	*Reps*	*m/s*	*Rest*	*RIR*
Olympic derivatives	5	4		120 - 180 sec	5 - 6	5	4		120 - 180 sec	5 - 6	5	4		120 - 180 sec	5 - 6	5	4		120 - 180 sec	5 - 6	5	4		120 - 180 sec	5 - 6
Lower body push	4	5	0.55-0.50	120 - 180 sec	4	4	4	0.50-0.45	120 - 180 sec	3	4	3	0.45-0.40	120 - 180 sec	2 - 3	4	5	0.55-0.50	120 - 180 sec	4	4	4	0.50-0.45	120 - 180 sec	3
Lower body push : split stance	4	5 l/r	0.55-0.50	120 - 180 sec	4	4	4 l/r	0.50-0.45	120 - 180 sec	3	4	3 l/r	0.45-0.40	120 - 180 sec	2 - 3	4	5 l/r	0.55-0.50	120 - 180 sec	4	4	4 l/r	0.50-0.45	120 - 180 sec	3
Lower body pull	3	5	0.60-0.55	0 sec	4	3	4	0.55-0.50	0 sec	3	3	3	0.50-0.45	0 sec	2 - 3	3	5	0.60-0.55	0 sec	4	3	4	0.55-0.50	0 sec	3
Upper body pull	3	5		120 - 180 sec	4	3	4		120 - 180 sec	3	3	3		120 - 180 sec	2 - 3	3	5		120 - 180 sec	4	3	4		120 - 180 sec	3
Accessory Exercise																									
Anti-lateral flexion	3	20 sec l/r		0 sec		3	25 sec l/r		0 sec		3	30 sec l/r		0 sec		3	25 sec l/r		0 sec		3	30 sec l/r		0 sec	
Rotation	3	8 l/r		60 sec	4	3	8 l/r		60 sec	3	3	8 l/r		60 sec	3	3	8 l/r		60 sec		3	8 l/r		60 sec	

Day 2	week 1					week 2					week 3					week 4					week 5				
Main Exercise	*Set*	*Reps*	*m/s*	*Rest*	*RIR*	*Set*	*Reps*	*m/s*	*Rest*	*RIR*	*Set*	*Reps*	*m/s*	*Rest*	*RIR*	*Set*	*Reps*	*m/s*	*Rest*	*RIR*	*Set*	*Reps*	*m/s*	*Rest*	*RIR*
Olympic derivatives	5	4 (2 2)		120 - 180 sec	5 - 6	5	4 (2 2)		120 - 180 sec	5 - 6	5	4 (2 2)		120 - 180 sec	5 - 6	5	4 (2 2)		120 - 180 sec	5 - 6	5	4 (2 2)		120 - 180 sec	5 - 6
Lower body push	4	5	0.55-0.50	120 - 180 sec	4	4	4	0.50-0.45	120 - 180 sec	3	4	3	0.45-0.40	120 - 180 sec	2 - 3	4	5	0.55-0.50	120 - 180 sec	4	4	4	0.50-0.45	120 - 180 sec	3
Lower body push : split stance	4	5 l/r	0.55-0.50	120 - 180 sec	4	4	4 l/r	0.50-0.45	120 - 180 sec	3	4	3 l/r	0.45-0.40	120 - 180 sec	2 - 3	4	5 l/r	0.55-0.50	120 - 180 sec	4	4	4 l/r	0.50-0.45	120 - 180 sec	3
Lower body pull: hip (1 leg)	3	5 l/r		0 sec	4	3	4 l/r		0 sec	3	3	3 l/r		0 sec	2 - 3	3	5 l/r		0 sec	4	3	4 l/r		0 sec	3
Upper body pull	3	5		120 - 180 sec	4	3	4		120 - 180 sec	3	3	3		120 - 180 sec	2 - 3	3	5		120 - 180 sec	4	3	4		120 - 180 sec	3
Accessory Exercise																									
Anti-extension	3	8		0 sec	4	3	8		0 sec	3	3	8		0 sec	2 - 3	3	8		0 sec	4	3	8		0 sec	3
Anti-flexion	3	8		60 sec	4	3	8		60 sec	3	3	8		60 sec	2 - 3	3	8		60 sec	4	3	8		60 sec	3

Power phase 1

Day 1	week 1					week 2					week 3					week 4					week 5					week 6					week 7				
Main Exercise	*Set*	*Reps*	*m/s*	*Rest*	*RIR*	*Set*	*Reps*	*m/s*	*Rest*	*RIR*	*Set*	*Reps*	*m/s*	*Rest*	*RIR*	*Set*	*Reps*	*m/s*	*Rest*	*RIR*	*Set*	*Reps*	*m/s*	*Rest*	*RIR*	*Set*	*Reps*	*m/s*	*Rest*	*RIR*	*Set*	*Reps*	*m/s*	*Rest*	*RIR*
Olympic lift	3	4 3 2		120 - 180 sec	5	3	4 3 2		120 - 180 sec	4	4	4 3 2 1		120 - 180 sec	3	3	4 3 2		120 - 180 sec	5	3	4 3 2		120 - 180 sec	4	4	4 3 2 1		120 - 180 sec	3	3	3 2 1		120 - 180 sec	5
Lower body push : ballistic	4	5	1.0-1.2	0 sec	5	4	4	0.9-1.1	0 sec	4	4	3	0.8-1.0	0 sec	3	4	5	1.0-1.2	0 sec	5	4	4	0.9-1.1	0 sec	4	4	3	0.8-1.0	0 sec	3	3	5	1.0-1.2	0 sec	5
Lower body plyometric	4	3		120 - 180 sec		4	3		120 - 180 sec		4	3		120 - 180 sec		4	3		120 - 180 sec		4	3		120 - 180 sec		4	3		120 - 180 sec		3	3		120 - 180 sec	
Lower body push : 1 leg	3	4 l/r		0 sec	5	3	3 l/r		0 sec	4	3	2 lr		0 sec	3	3	4 l/r		0 sec	5	3	3 l/r		0 sec	4	3	2 lr		0 sec	3	2	4 l/r		0 sec	5
Lower body plyometric	3	2 l/r		120 - 180 sec		3	2 l/r		120 - 180 sec		3	2 l/r		120 - 180 sec		3	2 l/r		120 - 180 sec		3	2 l/r		120 - 180 sec		3	2 l/r		120 - 180 sec		2	2 l/r		120 - 180 sec	
Accessory Exercise																																			
Lower body pull : knee	2	4		0 sec	3	2	5		0 sec	2	2	6		0 sec	1	2	4		0 sec	3	2	5		0 sec	2	2	6		0 sec	1	2	4		0 sec	3
Anti-lateral flexion	3	20 sec l/r		0 sec		3	25 sec l/r		0 sec		3	30 sec l/r		0 sec		3	20 sec l/r		0 sec		3	25 sec l/r		0 sec		3	30 sec l/r		0 sec		2	20 sec l/r		0 sec	
Rotation	3	8 l/r		60 sec	5	3	8 l/r		60 sec	4	3	8 l/r		60 sec	3	3	8 l/r		60 sec	5	3	8 l/r		60 sec	4	3	8 l/r		60 sec	3	2	8 l/r		60 sec	5

Day 2	week 1					week 2					week 3					week 4					week 5					week 6					week 7				
Main Exercise	*Set*	*Reps*	*m/s*	*Rest*	*RIR*	*Set*	*Reps*	*m/s*	*Rest*	*RIR*	*Set*	*Reps*	*m/s*	*Rest*	*RIR*	*Set*	*Reps*	*m/s*	*Rest*	*RIR*	*Set*	*Reps*	*m/s*	*Rest*	*RIR*	*Set*	*Reps*	*m/s*	*Rest*	*RIR*	*Set*	*Reps*	*m/s*	*Rest*	*RIR*
Olympic lift	3	4 3 2		120 - 180 sec	5	3	4 3 2		120 - 180 sec	4	4	4 3 2 1		120 - 180 sec	3	3	4 3 2		120 - 180 sec	5	3	4 3 2		120 - 180 sec	4	4	4 3 2 1		120 - 180 sec	3	3	3 2 1		120 - 180 sec	5
Lower body push	4	5	1.0-0.9	0 sec	5	4	4	0.9-0.8	0 sec	4	4	3	0.8-0.7	0 sec	3	4	5	1.0-0.9	0 sec	5	4	4	0.9-0.8	0 sec	4	4	3	0.8-0.7	0 sec	3	3	5	1.0-0.9	0 sec	5
Lower body plyometric	4	2 l/r		120 - 180 sec		4	2 l/r		120 - 180 sec		4	2 l/r		120 - 180 sec		4	2 l/r		120 - 180 sec		4	2 l/r		120 - 180 sec		4	2 l/r		120 - 180 sec		3	2 l/r		120 - 180 sec	
Lower body push : 1 leg	3	4 l/r	1.0-0.9	0 sec	5	3	3 l/r	0.9-0.8	0 sec	4	3	2 lr	0.8-0.7	0 sec	3	3	4 l/r	1.0-0.9	0 sec	5	3	3 l/r	0.9-0.8	0 sec	4	3	2 lr	0.8-0.7	0 sec	3	2	4 l/r	1.0-0.9	0 sec	5
Lower body plyometric	3	2 l/r		120 - 180 sec		3	2 l/r		120 - 180 sec		3	2 l/r		120 - 180 sec		3	2 l/r		120 - 180 sec		3	2 l/r		120 - 180 sec		3	2 l/r		120 - 180 sec		2	2 l/r		120 - 180 sec	
Accessory Exercise																																			
Lower body pull : hip	2	7		0 sec	5	2	6		120 - 180 sec	4	2	5		120 - 180 sec	3	2	7		0 sec	5	2	6		120 - 180 sec	4	2	5		120 - 180 sec	3	2	7		120 - 180 sec	5
Anti-extension	3	8		0 sec	5	3	8		0 sec	4	3	8		0 sec	3	3	8		0 sec	5	3	8		0 sec	4	3	8		0 sec	3	2	20 sec l/r		0 sec	5
Rotation	3	8 l/r		60 sec	5	3	8 l/r		60 sec	4	3	8 l/r		60 sec	3	3	8 l/r		60 sec	5	3	8 l/r		60 sec	4	3	8 l/r		60 sec	3	2	8 l/r		60 sec	5

Strength phase 2

Day 1	week 1					week 2					week 3					week 4					week 5					week 6				
Main Exercise	*Set*	*Reps*	*m/s*	*Rest*	*RIR*	*Set*	*Reps*	*m/s*	*Rest*	*RIR*	*Set*	*Reps*	*m/s*	*Rest*	*RIR*	*Set*	*Reps*	*m/s*	*Rest*	*RIR*	*Set*	*Reps*	*m/s*	*Rest*	*RIR*	*Set*	*Reps*	*m/s*	*Rest*	*RIR*
Olympic lift	4	4 3 2 1		120 - 180 sec	4	4	3 3 2 1		120 - 180 sec	3	4	3 2 2 1		120 - 180 sec	2	4	4 3 2 1		120 - 180 sec	4	4	3 3 2 1		120 - 180 sec	3	4	3 2 2 1		120 - 180 sec	1 - 2
Lower body push	3	2 2 1	0.50-0.40	30 sec	4	3	2 1 1	0.45-0.35	30 sec	3	3	1 1 1	0.40-0.30	30 sec	2	3	2 2 1	0.50-0.40	30 sec	4	3	2 1 1	0.45-0.35	30 sec	3	3	1 1 1	0.40-0.30	30 sec	1 - 2
Lower body plyometric	3	3		120 - 180 sec		3	3		120 - 180 sec		3	3		120 - 180 sec		3	3		120 - 180 sec		3	3		120 - 180 sec		3	3		120 - 180 sec	
Lower body push : 1 leg	3	4 l/r	0.70-0.80	0 sec	4	3	4 l/r	0.80-0.90	0 sec	3	3	4 l/r	0.90-1.0	0 sec	2	3	4 l/r	0.70-0.80	0 sec	4	3	4 l/r	0.80-0.90	0 sec	3	3	4 l/r	0.90-1.0	0 sec	1 - 2
Lower body plyometric	3	3 l/r		120 - 180 sec		3	3 l/r		120 - 180 sec		3	3 l/r		120 - 180 sec		3	3 l/r		120 - 180 sec		3	3 l/r		120 - 180 sec		3	3 l/r		120 - 180 sec	
Accessory Exercise																														
Lower body pull : knee	2	3		0 sec	3	2	4		0 sec	2	2	5		0 sec	1	2	3		0 sec	3	2	4		0 sec	2	2	5		0 sec	1
Anti-lateral flexion	3	10 l/r		60 sec		3	8 l/r		60 sec		3	8 l/r		60 sec		3	10 l/r		0 sec		3	8 l/r		60 sec		3	8 l/r		60 sec	
Rotation	3	6 l/r		60 sec	4	3	6 l/r		60 sec	3	3	6 l/r		60 sec	2	3	6 l/r		60 sec	4	3	6 l/r		60 sec	3	3	6 l/r		60 sec	2

Day 2	week 1					week 2					week 3					week 4					week 5					week 6				
Main Exercise	*Set*	*Reps*	*m/s*	*Rest*	*RIR*	*Set*	*Reps*	*m/s*	*Rest*	*RIR*	*Set*	*Reps*	*m/s*	*Rest*	*RIR*	*Set*	*Reps*	*m/s*	*Rest*	*RIR*	*Set*	*Reps*	*m/s*	*Rest*	*RIR*	*Set*	*Reps*	*m/s*	*Rest*	*RIR*
Olympic lift	4	4 3 2 1		120 - 180 sec	4	4	3 3 2 1		120 - 180 sec	3	4	3 2 2 1		120 - 180 sec	2	4	4 3 2 1		120 - 180 sec	4	4	3 3 2 1		120 - 180 sec	3	4	3 2 2 1		120 - 180 sec	1 - 2
Lower body push : split stance	3	5 l/r	0.50-0.40	30 sec	4	3	4 l/r	0.45-0.35	30 sec	3	3	3 l/r	0.40-0.30	30 sec	2	3	5 l/r	0.50-0.40	30 sec	4	3	4 l/r	0.45-0.35	30 sec	3	3	3 l/r	0.40-0.30	30 sec	1 - 2
Lower body plyometric	3	2 l/r		120 - 180 sec		3	2 l/r		120 - 180 sec		3	2 l/r		120 - 180 sec		3	2 l/r		120 - 180 sec		3	2 l/r		120 - 180 sec		3	2 l/r		120 - 180 sec	
Lower body ballistic	3	4	1.0-1.2	0 sec	4	3	4	1.0-1.2	0 sec	3	3	4	1.0-1.2	0 sec	2	3	4	1.0-1.2	0 sec	4	3	4	1.0-1.2	0 sec	3	3	4	1.0-1.2	0 sec	1 - 2
lower body plyometric	3	3		120 - 180 sec		3	3		120 - 180 sec		3	3		120 - 180 sec		3	3		120 - 180 sec		3	3		120 - 180 sec		3	3		120 - 180 sec	
Accessory Exercise																														
Lower body pull: hip (1 leg)	2	7 l/r		0 sec	3	2	6 l/r		0 sec	2	2	5 l/r		0 sec	1	2	7 l/r		0 sec	3	2	6 l/r		0 sec	2	2	5 l/r		0 sec	1
Anti-extension	3	30 sec		60 sec		3	35 sec		60 sec		3	40 sec		60 sec		3	30 sec		0 sec		3	35 sec		60 sec		3	40 sec		60 sec	
Rotation	3	10 l/r		60 sec	4	3	10 l/r		60 sec	3	3	10 l/r		60 sec	2	3	10 l/r		60 sec	4	3	10 l/r		60 sec	3	3	10 l/r		60 sec	2

Power phase 2

Day 1	week 1					week 2					week 3					week 4					week 5				
Main Exercise	*Set*	*Reps*	*m/s*	*Rest*	*RIR*	*Set*	*Reps*	*m/s*	*Rest*	*RIR*	*Set*	*Reps*	*m/s*	*Rest*	*RIR*	*Set*	*Reps*	*m/s*	*Rest*	*RIR*	*Set*	*Reps*	*m/s*	*Rest*	*RIR*
Olympic lift	4	2		120 - 180 sec	4	4	2 2 2 1		120 - 180 sec	3	4	2 2 1 1		120 - 180 sec	2	4	2 1 1 1		120 - 180 sec	1 - 2	4	1		120 - 180 sec	1 - 2
Lower body push	4	3	0.6-0.7	0 sec	4	4	3	0.7-0.8	0 sec	3	4	3	0.8-0.9	0 sec	2	4	3	0.9-1.0	0 sec	1 - 2	4	3	1.0-1.2	0 sec	1 - 2
Lower body ballistic	4	3	>1.2	120 - 180 sec		4	3	>1.2	120 - 180 sec		4	3	>1.2	120 - 180 sec		4	3	>1.2	120 - 180 sec		4	3	>1.2	120 - 180 sec	
Lower body push: split stance	3	5 l/r	0.60-0.50	0 sec	4	3	4 l/r	0.55-0.45	0 sec	3	3	3 l/r	0.40-0.30	0 sec	2	3	2 l/r	0.35-0.25	0 sec	1 - 2	3	2 l/r	0.35-0.25	0 sec	1 - 2
Lower body plyometric	3	2 l/r	>1.2	120 - 180 sec		3	2 l/r	>1.2	120 - 180 sec		3	2 l/r	>1.2	120 - 180 sec		3	2 l/r	>1.2	120 - 180 sec		3	2 l/r	>1.2	120 - 180 sec	
Accessory Exercise																									
Lower body pull: hip (1 leg)	2	4 l/r		0 sec	4	2	4 l/r		0 sec	4	2	4 l/r		0 sec	4	2	4 l/r		0 sec	4	2	4 l/r		0 sec	4
Anti-lateral flexion	3	8 l/r		0 sec		3	8 l/r		0 sec		3	8 l/r		0 sec		3	8 l/r		0 sec		3	8 l/r		0 sec	
Anti-flexion	3	40 sec		60 sec		3	45 sec		60 sec		3	50 sec		60 sec		3	55 sec		60 sec		3	60 sec		60 sec	

Day 2	week 1					week 2					week 3					week 4					week 5				
Main Exercise	*Set*	*Reps*	*m/s*	*Rest*	*RIR*	*Set*	*Reps*	*m/s*	*Rest*	*RIR*	*Set*	*Reps*	*m/s*	*Rest*	*RIR*	*Set*	*Reps*	*m/s*	*Rest*	*RIR*	*Set*	*Reps*	*m/s*	*Rest*	*RIR*
Olympic lift	4	2		120 - 180 sec	4	4	2 2 2 1		120 - 180 sec	3	4	2 2 1 1		120 - 180 sec	2	4	2 1 1 1		120 - 180 sec	1 - 2	4	1		120 - 180 sec	1 - 2
Lower body push	4	3	0.60-0.50	0 sec	4	4	3 3 2 2	0.55-0.45	0 sec	3	4	3 2 2 2	0.40-0.30	0 sec	2	4	2	0.35-0.25	0 sec	1 - 2	4	2 2 1 1	0.35-0.25	0 sec	1 - 2
Lower body plyometric	4	3		120 - 180 sec		4	3		120 - 180 sec		4	3		120 - 180 sec		4	3		120 - 180 sec		4	3		120 - 180 sec	
Lower body push : 1 leg	3	5 l/r		0 sec	4	3	5 l/r		0 sec	3	3	5 l/r		0 sec	2	3	5 l/r		0 sec	1 - 2	3	5 l/r		0 sec	1 - 2
Lower body plyometric	3	3 l/r		120 - 180 sec		3	3 l/r		120 - 180 sec		3	3 l/r		120 - 180 sec		3	3 l/r		120 - 180 sec		3	3 l/r		120 - 180 sec	
Accessory Exercise																									
Lower body pull: hip (1 leg)	2	4 l/r		0 sec	4	2	4 l/r		0 sec	4	2	4 l/r		0 sec	4	2	4 l/r		0 sec	4	2	4 l/r		0 sec	4
Anti-lateral flexion	2	20 sec l/r		0 sec		2	25 sec l/r		0 sec		2	30 sec l/r		0 sec		2	35 sec l/r		0 sec		2	40 sec l/r		0 sec	
Rotation	2	6 l/r		60 sec	4	2	6 l/r		60 sec	4	2	6 l/r		60 sec	4	2	6 l/r		60 sec	4	2	6 l/r		60 sec	4

Power phase 3

Day 1	week 1					week 2					week 3					week 4					week 5				
Main Exercise	*Set*	*Reps*	*m/s*	*Rest*	*RIR*	*Set*	*Reps*	*m/s*	*Rest*	*RIR*	*Set*	*Reps*	*m/s*	*Rest*	*RIR*	*Set*	*Reps*	*m/s*	*Rest*	*RIR*	*Set*	*Reps*	*m/s*	*Rest*	*RIR*
Olympic lift	5	2 2 1 2 2	peak > 1.8	120 - 180 sec		5	2 2 1 2 2	peak > 1.9	120 - 180 sec		5	2 2 1 2 2	peak > 2.0	120 - 180 sec		5	2 2 1 2 2	peak > 1.9	120 - 180 sec		5	2 2 1 2 2	peak > 2.0	120 - 180 sec	
Lower body push	3	2 2 1	0.50-0.40	30 sec	4	3	2 1 1	0.45-0.35	30 sec	3	3	1 1 1	0.40-0.30	30 sec	2	3	2 2 1	0.45-0.35	30 sec	3	3	2 1 1	0.40-0.30	30 sec	2
Lower body plyometric	3	2 lr		120 - 180 sec		3	2 lr		120 - 180 sec		3	2 lr		120 - 180 sec		3	2 lr		120 - 180 sec		3	2 lr		120 - 180 sec	
Lower body push : 1 leg	3	5 l/r		0 sec	4	3	5 l/r		0 sec	3	3	5 l/r		0 sec	2	3	5 l/r		0 sec	3	3	5 l/r		0 sec	2
Lower body plyometric	3	2 l/r		120 - 180 sec		3	2 l/r		120 - 180 sec		3	2 l/r		120 - 180 sec		3	2 l/r		120 - 180 sec		3	2 l/r		120 - 180 sec	
Accessory Exercise																									
Lower body pull: hip (1 leg)	2	4 l/r		0 sec	4	2	4 l/r		0 sec	4	2	4 l/r		0 sec	4	2	4 l/r		0 sec	4	2	4 l/r		0 sec	4
Anti-lateral flexion	3	8 l/r		0 sec		3	8 l/r		0 sec		3	8 l/r		0 sec		3	8 l/r		0 sec		3	8 l/r		0 sec	
Anti-rotation	3	8 l/r		60 sec	4	3	8 l/r		60 sec	4	3	8 l/r		60 sec	4	3	8 l/r		60 sec	4	3	8 l/r		60 sec	4

Day 2	week 1					week 2					week 3					week 4					week 5				
Main Exercise	*Set*	*Reps*	*m/s*	*Rest*	*RIR*	*Set*	*Reps*	*m/s*	*Rest*	*RIR*	*Set*	*Reps*	*m/s*	*Rest*	*RIR*	*Set*	*Reps*	*m/s*	*Rest*	*RIR*	*Set*	*Reps*	*m/s*	*Rest*	*RIR*
Olympic lift	5	2 2 1 2 2	peak > 1.8	120 - 180 sec		5	2 2 1 2 2	peak > 1.9	120 - 180 sec		5	2 2 1 2 2	peak > 2.0	120 - 180 sec		5	2 2 1 2 2	peak > 1.9	120 - 180 sec		5	2 2 1 2 2	peak > 2.0	120 - 180 sec	
Lower body push	3	2 2 1	0.50-0.40	30 sec	4	3	2 1 1	0.45-0.35	30 sec	3	3	1 1 1	0.40-0.30	30 sec	2	3	2 2 1	0.45-0.35	30 sec	3	3	2 1 1	0.40-0.30	30 sec	2
Lower body plyometric	3	3		120 - 180 sec		3	3		120 - 180 sec		3	3		120 - 180 sec		3	3		120 - 180 sec		3	3		120 - 180 sec	
Lower body push : split stance	3	5 l/r	0.50-0.40	0 sec	4	3	4 l/r	0.45-0.35	0 sec	3	3	3 l/r	0.40-0.30	0 sec	2	3	4 l/r	0.45-0.35	0 sec	3	3	3 l/r	0.40-0.30	0 sec	2
Lower body plyometric	3	3 l/r		120 - 180 sec		3	3 l/r		120 - 180 sec		3	3 l/r		120 - 180 sec		3	3 l/r		120 - 180 sec		3	3 l/r		120 - 180 sec	
Accessory Exercise																									
Lower body pull: hip (1 leg)	2	4 l/r		0 sec	4	2	4 l/r		0 sec	4	2	4 l/r		0 sec	4	2	4 l/r		0 sec	4	2	4 l/r		0 sec	4
Rotation	2	8 l/r		0 sec	4	2	8 l/r		0 sec	4	2	8 l/r		0 sec	4	2	8 l/r		0 sec	4	2	8 l/r		0 sec	4
Anti-extension	2	10 sec l/r		60 sec		2	15 sec l/r		60 sec		2	20 sec l/r		60 sec		2	15 sec l/r		60 sec		2	20 sec l/r		60 sec	

Power phase 4

Day 1	week 1					week 2					week 3					week 4					week 5				
Main Exercise	*Set*	*Reps*	*m/s*	*Rest*	*RIR*	*Set*	*Reps*	*m/s*	*Rest*	*RIR*	*Set*	*Reps*	*m/s*	*Rest*	*RIR*	*Set*	*Reps*	*m/s*	*Rest*	*RIR*	*Set*	*Reps*	*m/s*	*Rest*	*RIR*
Olympic lift	4	2	peak > 1.8	120 - 180 sec		4	2 2 2 1	peak > 1.9	120 - 180 sec		4	2 2 1 1	peak > 2.0	120 - 180 sec		3	2 1 1	peak > 2.0	120 - 180 sec		3	1	peak > 2.0	120 - 180 sec	
Lower body push	3	2 2 1	0.50-0.40	30 sec	4	3	2 1 1	0.45-0.35	30 sec	3	3	1 1 1	0.40-0.30	30 sec	2	3	2 1 1	0.45-0.35	30 sec	3	3	1 1 1	0.40-0.30	30 sec	2
Lower body plyometric	3	2 lr		120 - 180 sec		3	2 lr		120 - 180 sec		3	2 lr		120 - 180 sec		3	2 lr		120 - 180 sec		3	2 lr		120 - 180 sec	
Lower body push : split stance	3	5 l/r	0.50-0.40	0 sec	4	3	4 l/r	0.45-0.35	0 sec	3	3	3 l/r	0.40-0.30	0 sec	2	3	4 l/r	0.45-0.35	0 sec	3	3	3 l/r	0.40-0.30	0 sec	2
Lower body plyometric	3	3 l/r		120 - 180 sec		3	3 l/r		120 - 180 sec		3	3 l/r		120 - 180 sec		3	3 l/r		120 - 180 sec		3	3 l/r		120 - 180 sec	

Day 2	week 1					week 2					week 3					week 4					week 5				
Main Exercise	*Set*	*Reps*	*m/s*	*Rest*	*RIR*	*Set*	*Reps*	*m/s*	*Rest*	*RIR*	*Set*	*Reps*	*m/s*	*Rest*	*RIR*	*Set*	*Reps*	*m/s*	*Rest*	*RIR*	*Set*	*Reps*	*m/s*	*Rest*	*RIR*
Olympic lift	4	2	peak > 1.8	120 - 180 sec		4	2 2 2 1	peak > 1.9	120 - 180 sec		4	2 2 1 1	peak > 2.0	120 - 180 sec		3	2 1 1	peak > 2.0	120 - 180 sec		3	1	peak > 2.0	120 - 180 sec	
Lower body push	3	2 2 1	0.50-0.40	30 sec	4	3	2 1 1	0.45-0.35	30 sec	3	3	1 1 1	0.40-0.30	30 sec	2	3	2 1 1	0.45-0.35	30 sec	3	3	1 1 1	0.40-0.30	30 sec	2
Lower body plyometric	3	3		120 - 180 sec		3	3		120 - 180 sec		3	3		120 - 180 sec		3	3		120 - 180 sec		3	3		120 - 180 sec	
Lower body push : 1 leg	3	5 l/r		0 sec	4	3	5 l/r		0 sec	4	3	5 l/r		0 sec	4	3	5 l/r		0 sec	4	3	5 l/r		0 sec	4
Lower body plyometric	3	2 l/r		120 - 180 sec		3	2 l/r		120 - 180 sec		3	2 l/r		120 - 180 sec		3	2 l/r		120 - 180 sec		3	2 l/r		120 - 180 sec	
